# Broadcast Spawning by *Pocillopora* Species on the Great Barrier Reef

**DOI:** 10.1371/journal.pone.0050847

**Published:** 2012-12-05

**Authors:** Sebastian Schmidt-Roach, Karen J. Miller, Erika Woolsey, Gabriele Gerlach, Andrew H. Baird

**Affiliations:** 1 Institute for Marine and Antarctic Studies, University of Tasmania, Hobart, Australia; 2 Australian Institute of Marine Science, Townsville, Queensland, Australia; 3 Australian Research Council Centre of Excellence for Coral Reef Studies, James Cook University, Townsville, Queensland, Australia; 4 Carl von Ossietzky University Oldenburg, Institute for Biology and Environmental Sciences, Oldenburg, Germany; University of New South Wales, Australia

## Abstract

The coral genus *Pocillopora* is one of the few to include some species that broadcast spawn gametes and some species that brood larvae, although reports of reproductive mode and timing vary within and among species across their range. Notably, the ubiquitous *Pocillopora damicornis* has been described as both a brooder and spawner, although evidence of broadcast spawning is rare. Here, we report observations of broadcast-spawning in four species of *Pocillopora* on the Great Barrier Reef (GBR), including *P. damicornis.* All species spawned predictably during the early morning, two days following the full moon, and spawning was observed in multiple months over the summer period (November to February). Eggs and sperm were free-spawned concurrently. Eggs were negatively buoyant and contained *Symbiodinium*. This newfound knowledge on the mode, timing and regularity of broadcast spawning in *Pocillopora* spp. on the GBR brings us one step closer to elucidating the complex reproductive ecology of these species.

## Introduction

Much conjecture exists about the reproductive biology of the coral genus *Pocillopora* despite it representing one of the most abundant and widely studied taxa of scleractinian corals. The genus is one of the few to include species that brood larvae (e.g. *P. damicornis*) and species that broadcast spawn gametes (e.g. *P. eydouxi*; [1]–[2]). Spawning in corals refers to the release of gametes into the water column for external fertilisation and larval development, whereas brooding refers to the development of planula larvae within the polyps [3]. Brooded planulae may originate from internal fertilization of eggs or from parthenogenesis [2]. The ecological and evolutionary consequences of such a diversity of reproductive modes within a single coral genus has been the subject of considerable research over the last decades (e.g. [4]–[5], [6]–[7]), although there still remain many gaps in our knowledge about when, and how, most *Pocillopora* spp. reproduce.

*Pocillopora damicornis* is thought to brood throughout most of its range (Table 1), and in Western Australia, Eastern Australia and Taiwan, molecular analysis indicate that brooded larvae are produced largely asexually [4]–[6], [7]–[8]. Other earlier examples of *Pocillopora* spp. brooding larvae have been discredited [3], except for one recent observation in the Philippines (*P. verrucosa*; [9]). In summary, of the seventeen formally accepted species of *Pocillopora* [10], three (*P. eydouxi, P. meandrina*, *P. elegans*) are broadcast spawners and two (*P. verrucosa* and *P. damicornis*) have a different mode of larval development among regions. In addition, some *P. damicornis* reproduce by brooding larvae and spawning gametes (Table 1).

**Table 1:** Reproductive mode of *Pocillopora* species (*inferred from histology).

Species	Location	Mode	Reference
*P. verrucosa*	Red Sea	Spawn*	[48]; [49]; [24]
	Maldives	Spawn*	[50]
	Okinawa	Spawn	[31], *ex situ*; [51]
	Philippines	Brood	[9]
	Red Sea	Spawn	[32], *in situ* &*ex situ*
*P. meandrina*	Hawaii	Spawn	[29], *in situ*; [30], *in situ*
	Enewetak (as *P. elegans*)	Brood	[52]
*P. eydouxi*	Okinawa	Spawn	[31], *ex situ*
	Hawaii	Spawn	[30], *in situ*
*P. damicornis*	Western Australia	Brood	[4] (asexual); [53]
	Western Australia	BroodandSpawn*	[5]
	Eastern Australia	Brood and Spawn*	[13] (spawning suggested based on the disappearance of eggs in histological samples)
	Eastern Pacific	Spawn*	[12]; [54]
	Eastern Australia	Brood	eg. [55]; [56]; [6] (asexual); [7] (asexual);
			*P. damicornis* Type α (asexual), Type β (asexual) and Type σ, [11]
	Thailand	Brood	[58]
	Taiwan	Brood	[9]; [8] (sexual and asexual)
	Hawaii, Enewetak	Brood	[59]
*P. elegans*	Eastern Pacific	Spawn*	[12]

At least some of the controversy around spatial variation in the reproductive mode of *Pocillopora* spp. is likely to be linked to the existence of cryptic species. For example, *P. damicornis* is now recognised to be a species complex rather than a single morphologically plastic species [11]. Of the five putative species within the *P. damicornis* complex, three were observed brooding (and at least two brood asexual larvae; Table 1). Spawning has been reported for *P.* cf. *damicornis* in the Eastern-Pacific [12], although evidence shows that this species actually resolves genetically within one clade with *P. verrucosa* and *P. damicornis* Type γ and thus is genetically distant to species observed brooding in Australia [11]. Clearly, the difficulties in distinguishing even among what are considered morphologically distinct species of *Pocillopora* has contributed to the conflicting reports on reproductive behaviour within species.

The mode of reproduction in *P. damicornis* is also a matter of conjecture. Like many species, spawning has never been observed in *P. damicornis* rather, it has been inferred from the disappearance of gametes in histological samples [5]–[12], [13]. In Australia, as in most other areas, all planula larvae examined appear to have been produced asexually [4]–[6], [7]–[11], however, the population genetic structure reflects random sexual reproduction with high genotypic diversity (e.g. [7]–[14]) suggesting important aspects of the life history of *P. damicornis* remain unknown. Here, we report the first observation of broadcast spawning of gametes in four *Pocillopora* species, including *P. damicornis*, and suggest that sexual reproduction is likely to occur regularly in pocilloporids on the GBR.

## Materials and Methods

All necessary permits were obtained for the described field studies. The samples were taken under Permit No G10/33440.1 issued to the Australian Institute for Marine Science by the Great Barrier Reef Marine Park Authority (GRBMPA) and Permit No 2011/158501 issued to Sebastian Schmidt-Roach by the Rottnest Island Authority.

**Figure 1:**
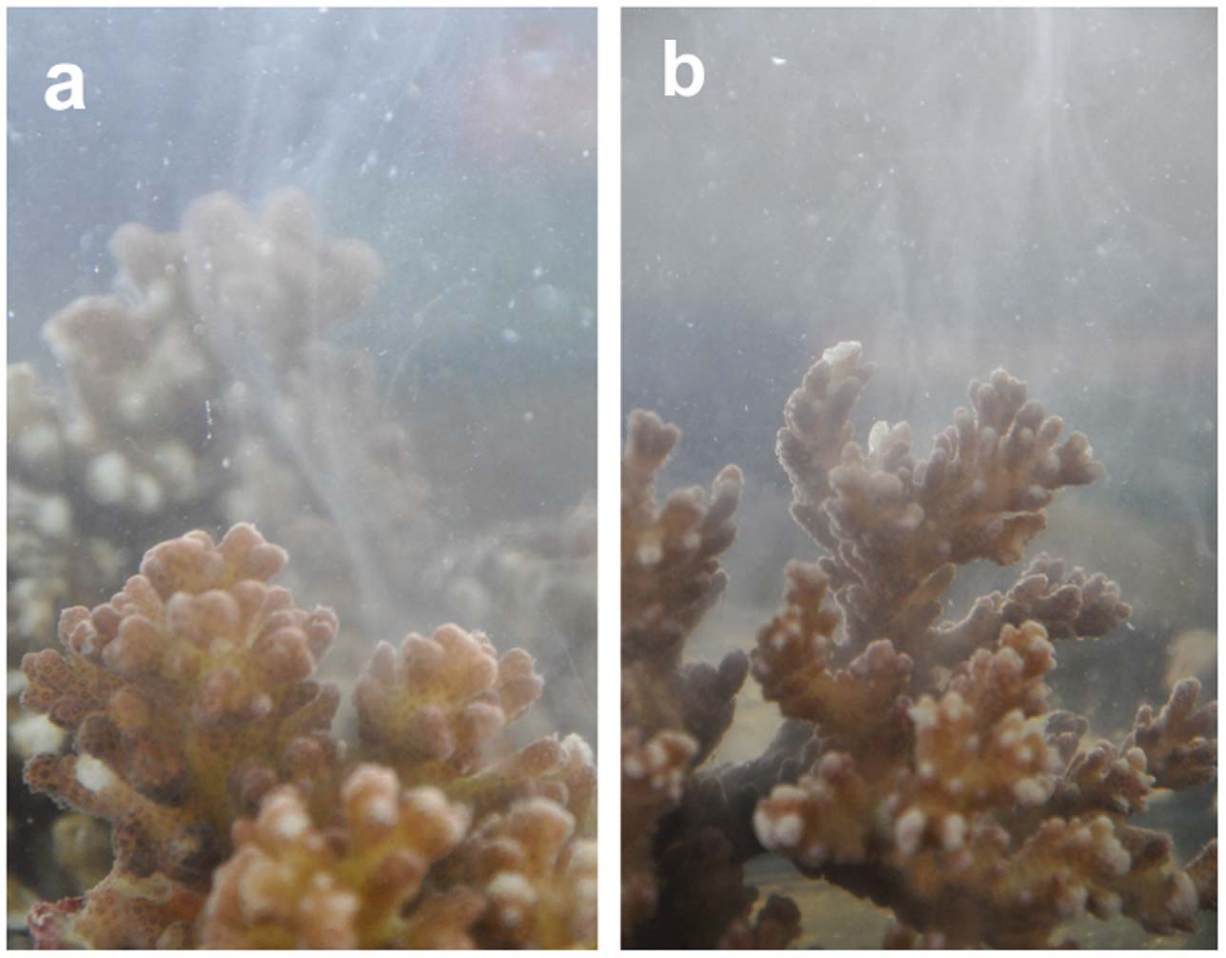
Sperm release by *Pocillopora damicornis*, One Tree Island.

Colonies of four *Pocillopora* species were collected 1–2 days before the full moon and maintained in a flow through seawater aquarium system at One Tree Island Research Station (23°30′29S; 152°4′37E) (*P. damicornis*) and Lizard Island Research Station (*P. eydouxi, P. verrucosa* and *P. meandrina*) (14°41′58S; 145°26′54E) in the summer of 2011/2012. The flow-through water was turned off around midnight every night for up to 20 days following full moon to enable spawning to be observed. In addition to specimens observed to spawn (a total of ten colonies; Table 2), at Lizard Island two colonies of *P. damicornis* Type α (*sensu* Schmidt-Roach et al [11]) and two specimens of *P. verrucosa* were isolated, but did not spawn. Specimens were visually identified and categorised according to Veron [10] and Schmidt-Roach et al. [11]. For a subset of specimens belonging to each morphotype, identification was further verified by sequencing of the mitochondrial ORF region [15] following protocols described in Schmidt-Roach et al. [11]. Furthermore, differences in the population ecology of *P. damicornis* from Western Australia (population structure predominantly asexual; [16]–[17]) and *P. damicornis* in Eastern Australia (population structure predominantly sexual; [18]–[19], [6]–[14], [7]) suggest these might be different species. Consequently, six specimens of *P. damicornis* from Rottnest Island, WA, were genotyped and sequences compared to existing data from the GBR. The alignment consisted of 11 sequences and 590 bp (NCBI accession numbers: JX983175-JX983186); reference sequences of previously identified cryptic species [11] were additionally included in the analysis to identify and illustrate the genealogical relationships amongst the taxa investigated in this study (NCBI accession numbers: JX985589; JX985612; JX985610; JX985592; JX985613; JX985605). Phylogenetic hypotheses were generated in MEGA4 [20] using the Neighbor-Joining algorithm under the JC correction and 100.000 bootstrap pseudo-replicated for nodal support [21]–[22].

**Figure 2:**
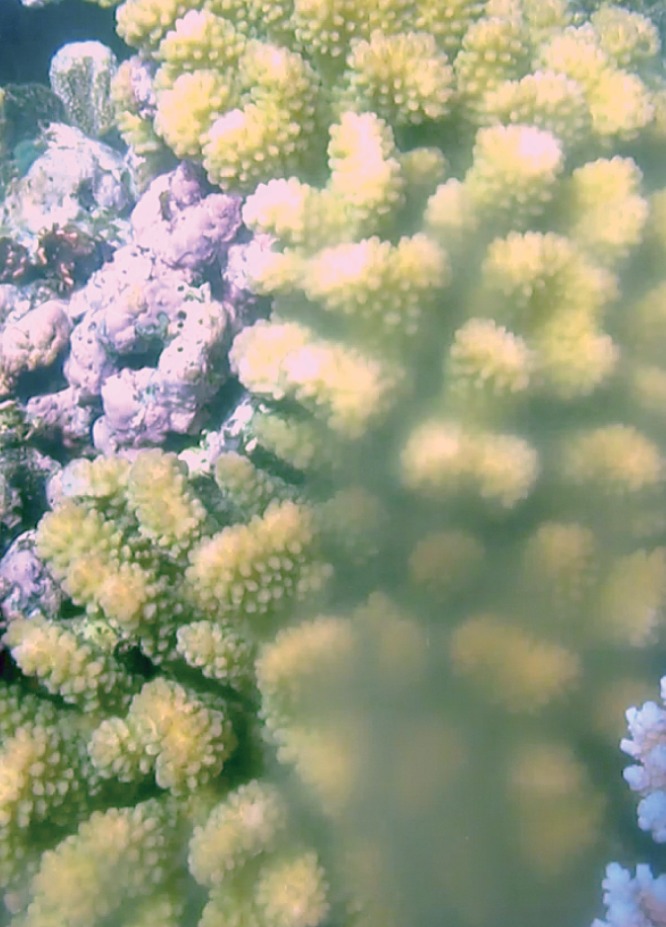
Spawning *Pocillopora meandrina* at Trimodal Reef, Lizard Island.

**Table 2:** Summary of spawning observations presented in this paper.

Dates of observation	Full moon	Location	Species (n)	Observation	Startat (hrs)	Sun-rise
12–13 Oct 2011	12 Oct 2011	One Tree Island	*P. damicornis* Type α (2)	Sperm released (*ex situ*) Fig. 1Brooded planulae (over night)	0610	0523/0522
11–12 Nov 2011	11 Nov 2011	LizardIsland	*P. meandrina* (1)	Spawn (*in situ*)	0625	0541
			*P. meandrina* (2)	Spawn (*ex situ*)		
			*P. eydouxi* (2)	Spawn (*ex situ*)		
			*P. verrucosa* (1)	Sperm released (*ex situ*) Fig. 2		
9 Feb 2012	8 Feb 2012	One Tree Island	*P. damicornis* Type α (2)	Spawning (*ex situ*)	∼ 0600–0700	0535

## Results and Discussion

**Figure 3:**
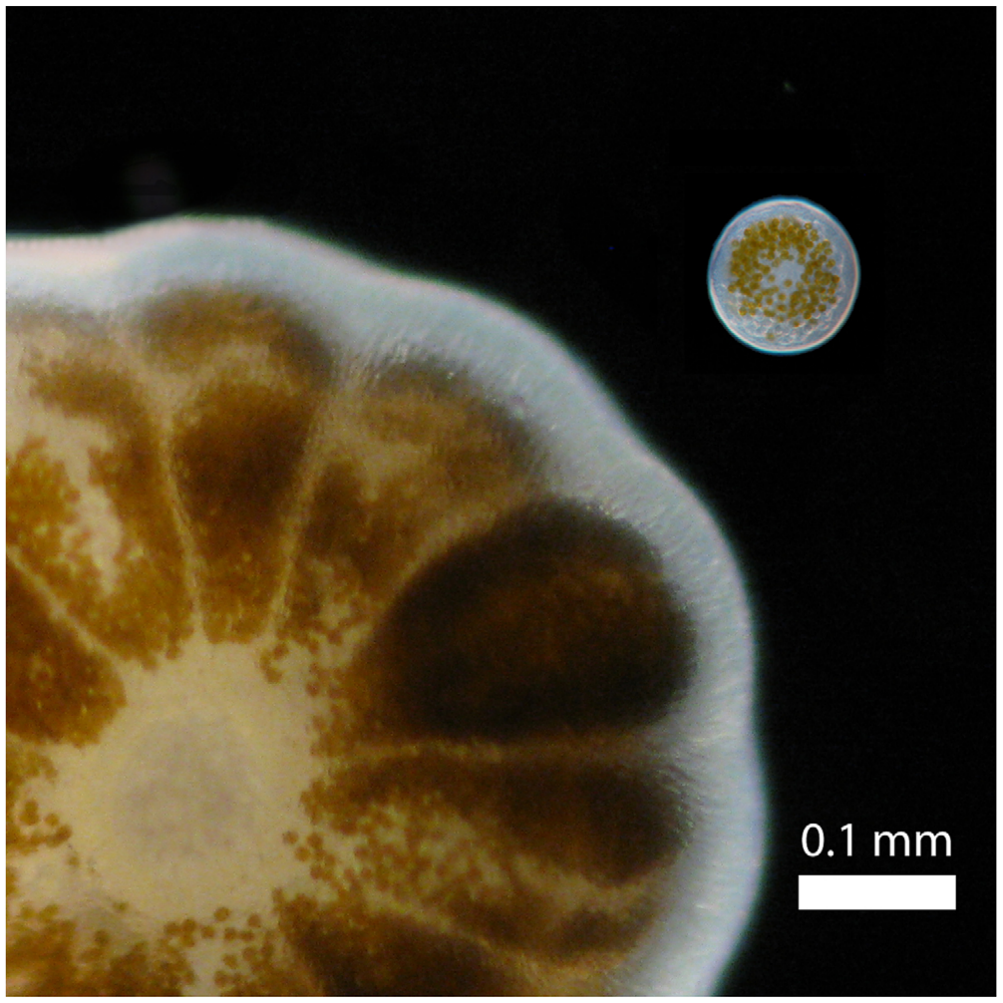
Brooded planula (*Pocillopora damicornis*, left) next to a spawned egg (*Pocillopora meandrina*, top right), indicating the size difference in *Pocillopora* between brooded and spawned offspring.

During the southern hemisphere summer of 2011/12 we observed gamete release in *Pocillopora eydouxi, P. verrucosa* and *P. meandrina* at Lizard Island, and *P. damicornis* at One Tree Island (Table 2); two locations at opposite ends of the GBR. Genotyping of 590 bp of the mitochondrial ORF region confirmed identifications based on morphology, except for *P. meandrina* and *P. eydouxi*, which share the same mitochondrial lineage [23], and therefore can not be distinguished by this marker. Spawning in all species occurred 1–2 days following the full moon, approximately 45 min after sunrise and continued for 2–3 hrs. Unlike most broadcast spawning coral species, *Pocillopora* gametes were free-spawned separately, rather than packaged in egg-sperm bundles. Sperm release was evident as a dense cloud surrounding the colony (Fig. 1 & 2). Due to the small size (see below), eggs were difficult to see, explaining why this behaviour may have been missed previously (e.g. [5]–[24]). The eggs were negatively buoyant, approximately 80 µm in diameter, and could easily be collected by siphoning the bottom of the aquaria below the colony. Eggs of *P. meandrina* (Fig. 3; Movie S1) and *P. eydouxi* contained algal symbionts, *Symbiodinium*. Ethanol preserved sperm samples of *P. damicornis* from One Tree Island also contained eggs. These eggs were 50–60 µm, which matches the size of mature *P. damicornis* eggs in histological sections [5]–[13] (Fig. S1). This strongly suggests the spawned eggs were mature rather than immature oocytes released due to handling. Fertilisation trials are required to confirm this unequivocally. Nevertheless, the release of sperm and mature eggs concurrently strongly suggests that sexual reproduction will occur. Numerous studies on sexual reproduction in other scleractinian corals (e.g. [25]–[26], [27]) have demonstrated that spawning behaviour in the laboratory is identical to that in the wild. Thus broadcast spawning of gametes with external fertilisation and larval development is likely to be the spawning behaviour in the field and the source of the sexual recruits of *P. damicornis* reported by previous studies (e.g. [6]).

Our observations are in agreement with reports from Hawaii [28]–[29], [30], Japan [31] and the Red Sea [32] regarding time and mode of larval development in these *Pocillopora* species, suggesting daytime spawning with a lunar periodicity may be characteristic for this genus across its range. Importantly, for *P. damicornis* this is the first direct observation of gamete release. In addition, brooded planulae were released the night before gamete release, indicating that both reproductive strategies occur simultaneously in the same colony supporting the inferences of previous histological studies [5]–[13]. Other coral species are known to vary their mode of reproduction in different geographic regions; however, *Goniastrea aspera* is the only other species in which individual colonies both brood and spawn [33].

**Figure 4:**
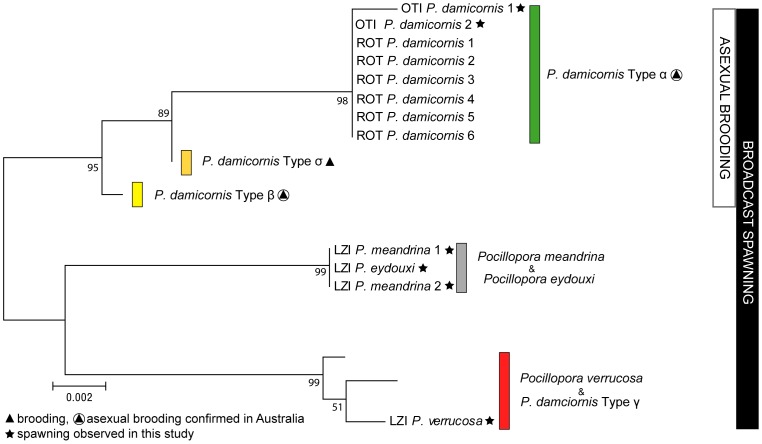
Mitochondrial phylogeny of *Pocillopora* specimens based on the ORF region. Coloured bars denote genetically distinct lineages or cryptic species identified by Schmidt-Roach et al [11]. *Pocillopora eydouxi* and *Pocillopora meandrina* shared identical mitochondrial haplotypes whilst *Pocillopora verrucosa* was recovered within the same clade with *P. damicornis* Type γ. *Pocillopora damicornis* Type σ and Type β were added in the phylogeny to indicate the close genetic relationship of brooding species within the genus *Pocillopora*. Black and white vertical bars indicate the proposed reproductive strategies of these taxa in Australia. Sample locations, indicated by three letter codes, are as follows: OTI = One Tree Island; ROT = Rottnest Island; LZI = Lizard Island. Numbers represent bootstrap values.

Histological studies have suggested that *P. damicornis* in Western Australia both spawns and broods (at Rottnest Island: [5]) and that the brooded larvae are generated asexually [4]. Sequence data confirmed that Western Australian specimens are genetically identical to *P. damicornis* Type α [11] on the east coast of Australia (Fig. 4). Our observations on the GBR of spawning in a lineage of *P. damicornis* known to brood, the release of brooded larvae and spawning over consecutive nights in the same colony, as well as the overwhelming evidence that brooded planulae are generated asexually [6], [7], [11] suggests the same is true of *P. damicornis* on the east coast of Australia. Consequently brooding lineages within *P. damicornis* throughout Australia most likely have a mixed mode of reproduction (Fig. 4). Indeed, the clonal generation of planulae seems to be characteristic of these lineages within *Pocillopora* [11] (Fig. 4), which contrasts with the sexual brooding reported in the sister genera *Stylophora* and *Seriatopora* [34]–[35], [36].

The evolutionary advantages of a mixed mode of asexual brooding and sexual spawning are still poorly understood. While settlement behavior and competency periods of brooded larvae of *P. damicornis* lineages are well studied (e.g. [37]–[38], [39]), nothing is known of the larval biology of spawned larvae in *Pocillopora*. Therefore, our observations represent an important foundation for future studies to further elucidate the differences between these larval types and the selective advantages of each mode of reproduction.

Typically for species with mixed modes of reproduction, asexual reproduction contributes to maintenance of local populations, with sexual progeny used for dispersal and recruitment to distant areas (i.e. the strawberry-coral model of Williams [40]). However, population genetic studies of *P. damicornis* on the GBR [18]–[19], [6]–[7] show only limited evidence of local recruitment of asexual planulae, but genetic subdivision even on relatively small spatial scales among populations suggests dispersal of sexual larvae is also limited. Thus there is little evidence that *P. damicornis* conforms to the predictions of the strawberry-coral model. It may be that the opposite occurs within *P. damicornis*, with the sexual progeny from broadcast spawning settling locally (as occurs for other broadcast spawning species – [19]–[41], [42]–[43]) and the larger, and potentially better-provisioned asexual larvae being more widely dispersed. Indeed, Richmond [37] reported that some brooded larvae of *P. damicornis* remained competent for over 100 days suggesting widespread dispersal of brooded larvae is possible. To date, population genetic studies have shown only limited evidence that asexual larvae of *P. damicornis* could be more widely dispersed, for example Ayre & Miller [6] found colonies with identical genotypes on opposite sides of One Tree Reef. If these corals represent recruitment from asexual planulae, then dispersal on the scale of kilometers may well occur.

Clearly further research is required to tease apart the roles of the two larval types in *P. damicornis* and their dispersal potential. Striking differences in size of the asexual (∼1000 µm) and sexual (∼80 µm) (Fig. 3) larvae suggest dispersal potential may well vary between them, although both types do contain zooxanthellae and therefore have the potential to be autotrophic [1]. Furthermore, the size difference raises questions of skeletal differences in early settlement between brooded and spawned larvae. While the skeletons of recruits of brooded offspring in this family are well studied [44] and often a focus of recruitment studies (e.g. [45]), the small size of spawned larvae may result in observable differences in size between sexual and asexual recruits and thus may enable the brooded and spawned recruits to be distinguished at settlement, similar to recruits in *Porites* spp. [46]. The predictable and consecutive spawning over several months that we report here makes *Pocillopora* ideal for future experiments to address such questions, as well as aspects of both the ecological and evolutionary processes in this important group of corals, including the maintenance of mixed mode of reproduction and hybridization in the genus *Pocillopora* [47–23], [11].

## Supporting Information

Figure S1 **Sperm and eggs of** ***Pocillopora damicornis*** **(after fixation in ethanol).**
(EPS)

Movie S1 **Spawning** ***Pocillopora meandrina*** **at Trimodal Reef, Lizard Island.**
(M4V)
